# Human Tau Isoforms and Proteolysis for Production of Toxic Tau Fragments in Neurodegeneration

**DOI:** 10.3389/fnins.2021.702788

**Published:** 2021-10-21

**Authors:** Ben Boyarko, Vivian Hook

**Affiliations:** ^1^Skaggs School of Pharmacy and Pharmaceutical Sciences, University of California, San Diego, La Jolla, CA, United States; ^2^Department of Neurosciences and Pharmacology, School of Medicine, University of California, San Diego, La Jolla, CA, United States

**Keywords:** tau isoforms, tau fragments, protease, mutations, neurotoxicity, tauopathies, Alzheimer’s disease, frontotemporal dementia

## Abstract

The human tau protein is implicated in a wide range of neurodegenerative “tauopathy” diseases, consisting of Alzheimer’s disease (AD) and frontotemporal lobar degeneration which includes progressive supranuclear palsy, corticobasal degeneration, Pick’s disease, and FTLD-tau (frontotemporal dementia with parkinsonism caused by MAPT mutations). Tau gene transcripts in the human brain undergo alternative splicing to yield 6 different tau protein isoforms that are expressed in different ratios in neurodegeneration which result in tau pathology of paired-helical filaments, neurofibrillary tangles, and tau fibrillar aggregates with detrimental microtubule destabilization. Protease-mediated tau truncation is an important post-translational modification (PTM) which drives neurodegeneration in a tau fragment-dependent manner. While numerous tau fragments have been identified, knowledge of the proteolytic steps that convert each parent tau isoform into specific truncated tau fragments has not yet been fully defined. An improved understanding of the relationships between tau isoforms and their proteolytic processing to generate neurotoxic tau fragments is important to the field. This review evaluates tau isoform expression patterns including PTMs and mutations that influence proteolysis of tau to generate toxic fragments that drive cognitive deficits in AD and other tauopathy models. This assessment identifies the gap in the field on understanding the details of proteolytic steps used to convert each tau isoform into fragments. Knowledge of the processing mechanisms of tau isoforms can lead to new protease targeted drug strategies to prevent the formation of toxic tau fragments in tauopathy neurodegenerative diseases.

## Introduction

Accumulation of tau in neurofibrillary tangles (NFTs) and fibrillar aggregates participates in dementia and behavioral dysfunctions in numerous neurodegenerative diseases which include Alzheimer’s disease (AD) and frontotemporal lobar degeneration which includes progressive supranuclear palsy (PSP), corticobasal degeneration (CBD), Pick’s disease (PiD), and FTLD-tau (frontotemporal dementia with parkinsonism caused by MAPT; Ballatore et al., 2007; Wolfe, 2012; Goedert et al., 2017). These brain disorders with tau pathogenesis are known as tauopathies. Human tau is a protein encoded by the microtubule associated protein tau (MAPT) gene on chromosome 17q21, which contains 14 exons (Martin et al., 2011; Caillet-Boudin et al., 2015; Goedert et al., 2017). Tau binds to microtubules (MT) and is essential for MT stabilization, assembly, and function in neuronal morphology and axonal transport (Weingarten et al., 1975; Cleveland et al., 1977; Ballatore et al., 2007). However, in tauopathies, tau loses its ability to bind to MTs through modifications of tau by phosphorylation (Alonso et al., 1994; Iqbal et al., 1994; Wang et al., 1995), proteolysis (Wang et al., 2007; Quinn et al., 2018), and other post-translational modifications (PTMs; Pevalova et al., 2006; Martin et al., 2011; Alquezar et al., 2020), as well as by tau mutations (Goedert and Spillantini, 2000, 2001; Spillantini and Goedert, 2000; Goedert, 2005; Goedert et al., 2017). The modified tau loses its MT stabilizing function and results in defective axonal transport (Kolarova et al., 2012; Combs et al., 2019). Modified tau dissociates from MTs and accumulates as NFTs or fibrillar aggregates resulting in disruption of cellular functions.

The molecular basis of aberrant tau forms that result in cellular dysfunctions, synaptic deficits, and cell death neurodegeneration has been an area of intense investigation. While mechanisms of tau phosphorylation have been studied extensively (Hanger et al., 2009; Wang et al., 2013; Šimić et al., 2016) less is known about the highly significant role of proteolysis of tau that generates numerous truncated fragments that drive neurodegeneration (Johnson, 2006; Quinn et al., 2018). The complete details of tau proteolysis to generate such fragments remains a gap in the field. For this reason, this review analyzes our current knowledge of the spectrum of tau fragments derived from multiple isoforms of tau to identify unanswered questions concerning the details of proteolytic steps utilized to convert each tau isoform into neurotoxic fragments. This assessment provides discussion of needed research in defining the spectrum of neurotoxic tau fragments specifically derived from each tau isoform by proteolysis. Complete knowledge of the proteolytic steps for producing neurotoxic tau fragments can lead to targeting of selected tau-cleaving proteases to inhibit production of highly toxic tau fragments in therapeutic strategies to alleviate tauopathy disease deficits.

## Tau Isoforms: Imbalance in Isoform Expression Patterns in Tauopathies

**FIGURE 1:**
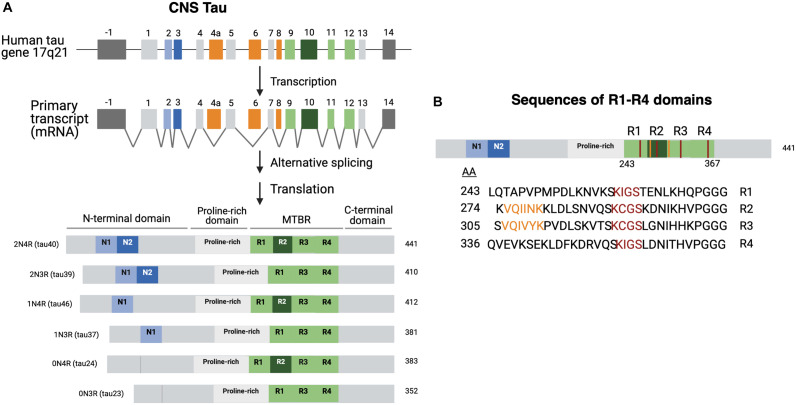
Tau isoforms generated through tau gene expression, alternative RNA splicing, and protein translation. **(A)** Tau biosynthesis in the CNS. Tau is found on chromosome 17 arm 21. The tau gene contains 16 exons. Exons 1, 4, 5, 7, 13 (light gray) and 9, 11, 12 (light green) are all constitutively transcribed in the CNS (Martin et al., 2011). Exons 9, 11, and 12 encode the R1, R3, and R4 domains, respectively (Martin et al., 2011; Goedert et al., 2017). Exon 10 encodes the R2 domain. Exon -1 is part of the promotor region and is not translated. Exons 4A, 6, and 8 (orange) are mostly expressed in peripheral tissues. Exon 14 is a part of the 3’ untranslated region of the mRNA sequence and is not translated (Goedert et al., 1989; Andreadis, 2005). Tau isoforms are generated by alternate splicing of exons 2 and 3 (light and dark blue) and exon 10 (dark green). The six isoforms range from 352 to 441 amino acids and can be referred to by the clone name as well. Structurally, tau is subdivided into the N-terminal domain, the proline rich domain, microtubule-binding domain region, and the C-terminal domain. **(B)** R domain MTBR primary sequences. The primary amino acid sequences of the microtubule binding region (MTBR) shows that R1 (243–273), R2 (274–304), R3 (305–335), and R4 (336–367) are partially repeated sequences (Barbier et al., 2019). 275 VQIINK 280 and 306 VQIVYK 311 are two motifs within R2 and R3 that have strong MT interactions and are important for intermolecular β-sheet formation (Moreno-Castillo et al., 2020).

There are predominantly six different isoforms generated by alternative mRNA splicing mechanisms (Ballatore et al., 2007; Goedert et al., 2017) which range from 45 to 65 kDa in length according to SDS gels (Liu and Gong, 2008; Figure 1A). Isoforms differ by the presence or absence of two 29 aa amino-terminal inserts encoded by exons 2 and 3 that are referred to as 0N, 1N, or 2N tau, and the presence or absence of the R2 domain, one of four partially repeated microtubule binding domain regions (MTBR) designated R1, R2, R3, and R4 (Figure 1A; Goedert et al., 1989, 2017; Barbier et al., 2019). Each R domain consists of four 31–32 similar and distinct amino acid motifs (Figure 1B), encoded by exons 9–12 with exon 10 encoding R2. Isoforms expressing R2 are referred as 4R and those lacking R2 are referred as 3R (Goedert et al., 1989; Barbier et al., 2019). Altogether the six isoforms are named as 0N3R, 0N4R, 1N3R, 1N4R, 2N3R, 2N4R, and are also referenced by the number of residues or clone name (Mandelkow and Mandelkow, 2012).

It is of interest that the ratio of 4R to 3R tau isoforms is approximately 1 in normal adult brain, but this ratio is differentially altered in neurodegenerative tauopathies, indicating an imbalance of 4R to 3R isoforms, regardless of whether the ratio shifts toward 4R or 3R (Gasparini et al., 2007). AD and frontotemporal dementia with parkinsonism, the non-Alzheimer’s tauopathy, are classified as mixed 3R and 4R tauopathies (Hong et al., 1998). Other tauopathies such as CBD and PiD are characterized by their expression of specific tau isoforms. PiD is a tauopathy primarily affected by 3R-tau accumulation into “Pick bodies” (Buée and Delacourte, 1999; Yoshida, 2006). The molecular basis for altered expression profiles of tau isoforms in different tauopathies is unknown. Therefore, investigation of regulatory mechanisms that control the biosynthesis of tau isoforms through tau gene transcription, RNA alternative splicing, and protein translation will advance understanding of the molecular basis for differential expression of tau isoforms among the different tauopathies.

Tau isoforms display differences in aggregation properties. Rates of assembly between 4R and 3R tau isoforms are distinct, with 4R tau isoforms assembling 2.5–3.0 times more rapidly compared to 3R tau isoforms, with no contributions by differences in amino-terminal insertions (Goedert and Jakes, 1990). These differences in assembly of 4R compared to 3R tau isoforms indicate variations in the kinetics of aggregation of 4R compared to 3R tau isoforms. Differences in aggregation properties of tau isoforms implicate heterogeneity in the compositions of tau aggregates among tauopathies expressing altered ratios of 4R and 3R tau isoforms.

Furthermore, tau isoform expression patterns and phosphorylation are developmentally regulated. The smallest tau isoform (0N3R) is the only form that is expressed in fetal human brain and is highly phosphorylated compared to adult tau (Goedert et al., 1993; Brion et al., 1994). A switch from 3R to 4R tau in mouse brain occurs between postnatal day 9 (P9) to P18 under the same time course as a conversion from high to low phosphorylation (Tuerde et al., 2018). It is noted that highly phosphorylated fetal tau is functional and does not polymerize into NFTs or fibrillar aggregates (Yu et al., 2009). This group compared individual phosphorylation sites of tau in the developing rat brain and pathological tau from human AD brain. It was found that phosphorylations at S202, T212, T231, S396, S404, and S422 were much higher in AD brain than in developing rat brain, whereas the phosphorylation levels of T181, S199, T205, S214, S262, S356, and S409 in AD brain were similar or lower than tau in the developing rat brain. This data suggests that different phosphorylation levels at selective sites of the tau protein may play a key role in converting tau from a biologically active molecule into one that inhibits MT assembly.

Studies investigating the regulation of tau phosphorylation have also been studied in human fetal and adult autopsy brain tissue (Hefti et al., 2019). Of the 20 fetal cases that showed immunoreactivity to at least one of the tau antibodies used, 18/20 (90%) showed positive staining for phosphorylation at S214 and 17/20 (85%) showed positive staining for PHF1 antibody (pS396, pS404). Only 4 cases were positive for AT8 staining of pS202 and pT205, and 2 for RZ3 staining of pT231. Four serine residues (S214, S396, S404, and S202) appeared to be phosphorylated in both fetal and AD cases. Interestingly, the investigators (Hefti et al., 2019) found phospho-tau positive aggregates in fetal human brain. These aggregates were positive to T214 and weakly immunoreactive to CP3 antibody (S202) and PHF-1. The aggregates appeared to be non-toxic as they were negative for thioflavin S staining, indicating that they were seed-incompetent.

It is not fully clear how the transition of the isoforms and phosphorylation are regulated, and further investigation is still needed. Moreover, it will be important to gain understanding of the propensity of distinct tau isoforms to become phosphorylated, the hallmark of neurodegeneration in tauopathies that occur in aging.

## Tau Pathology Involving Hyperphosphorylation

**FIGURE 2:**
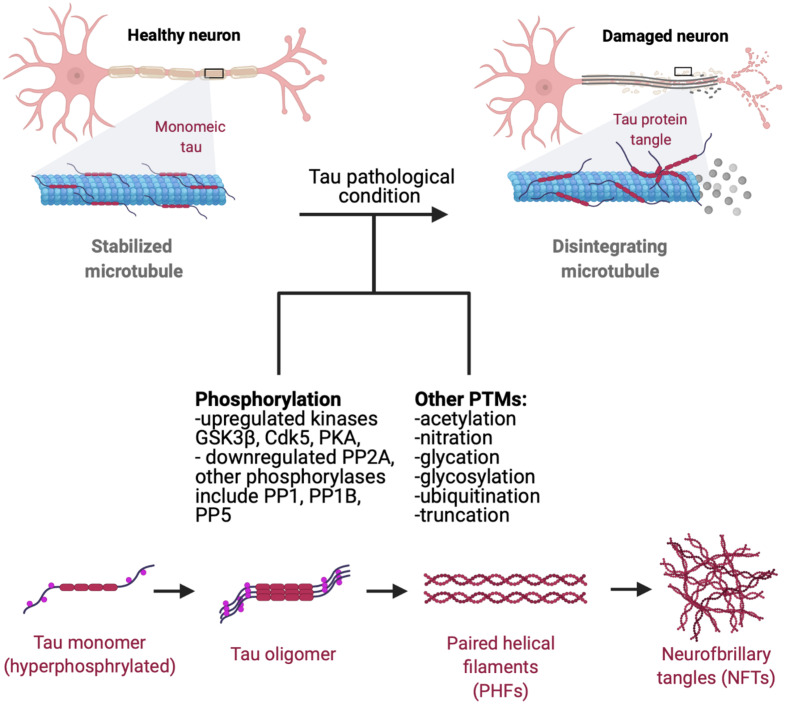
Progression of tau pathology. Microtubules are stabilized by monomeric tau through the MTBR (microtubule binding region). Hyperphosphorylation decreases tau stability and causes p-tau to dissociate from microtubules, leading to disintegration and disruption of normal axonal transport. Hyperphosphorylated tau gains affinity for other tau monomers, forming oligomeric species, adapting a β-sheet structure prior to NFT and aggregate formation (Maeda et al., 2006). Dissociated tau has a tendency to dimerize in an antiparallel fashion, self-assembling into dimers (Wille et al., 1992). These manifest later into PHFs and ultimately NFTs and fibrillar aggregates which can propagate and mediate cytotoxicity in neuronal cells. Phosphorylation of tau by GSK3β and Cdk5 affects tau-microtubule interactions by reducing tau’s microtubule affinity (Wagner et al., 1996), with protein kinase A (PKA) phosphorylation at S262 and S214 demonstrating similar effects (Biernat et al., 1993; Schneider et al., 1999). Recent studies have also shown that protein phosphatases (PP) PP1, PP2A, PP2B, and PP5 all dephosphorylate tau *in vitro*, with PP2A functioning as a major regulator of phosphorylation at multiple sites in the brain. PP2A is also partially down-regulated in AD brains in response to tubulin assembly (Sontag et al., 1999; Liu et al., 2005). Tau also goes a plethora of post-translational modifications such as acetylation, nitration, glycation, glycosylation, ubiquitination, and truncation, many of which are known to increase tau’s propensity to aggregate (Alquezar et al., 2020).

Tau pathology manifests as the deposition of insoluble aggregated NFTs, fibrillar aggregates, neuropil threads, paired helical fragments (PHFs), and straight filaments (ST) resulting from hyperphosphorylation of normal tau which leads to decreased tau affinity to MT (Figure 2; Wischik et al., 1988; Perry et al., 1991; Wille et al., 1992; Fitzpatrick et al., 2017). Upon self-assembly into PHF and STs, abnormally hyperphosphorylated cytosolic tau loses its ability to sequester normal microtubule associated proteins and disrupts microtubule assembly (Ballatore et al., 2007; Iqbal et al., 2010). Thus, it is a combination of loss-of-regular function consisting of detachment from MTs and loss of MT stabilization, as well as a toxic gain-of-function of PHF and NFT formation with derangement of normal cellular transport that contribute to tau pathology.

Interestingly, tau aggregates from AD brain compared to those from chronic traumatic encephalopathy display structural differences (Fitzpatrick et al., 2017; Falcon et al., 2019; Shi et al., 2021). Such findings suggest possible heterogeneity of tau aggregates and composition of tau fragments among different neurodegenerative disease conditions.

Significant spreading of tau pathology occurs in brain (Fuster-Matanzo et al., 2018; Franzmeier et al., 2020) and may involve tau secretion (Pernègre et al., 2019; Merezhko et al., 2020). Tau pathology and its propagation has been investigated in animal models of tauopathies. In transgenic mouse models overexpressing human tau P301L restricted to the entorhinal cortex (EC-II), tau pathology propagated through synaptic circuits to the dentate gyrus, CA fields of the hippocampus, and the cingulate cortex (de Calignon et al., 2012). P-tau aggregates also propagate to the frontal and temporal cortices as well as surrounding isocortex areas (Hu et al., 2017). Together, this data supports circuit-based, neuron-to-neuron tau transmission and correlates with the behavioral symptoms of AD including memory and cognitive deficits.

## Other Post Translational Modifications in the Context of Tau Pathology

Tau is a natively unfolded and intrinsically disordered protein that is remarkably resistant to hydrophobicity-driven collapse due to the lack of hydrophobic residues and is highly susceptible to aggregate within a wide range of pH values between 5 and 10 (Jeganathan et al., 2008). As such, in addition to phosphorylation, tau is especially prone to other forms of PTMs including (but not limited to) acetylation, ubiquitination, SUMOylation, glycation, and glycosylation (Park et al., 2018; Alquezar et al., 2020). It is of interest to understand the relationship of PTMs with tau phosphorylation in the regulation of toxic tau fragments and aggregation pathology.

Acetylation of mammalian proteins involves the N-terminal addition of acetyl groups from acetyl coenzyme A to lysine residues of polypeptide chains (Drazic et al., 2016). Acetylation of tau is enhanced in patients at early/moderate Braak states (Min et al., 2010). Disease and context specific abnormalities of HATs (histone acetyltransferase) and HDACs (histone deacetylases) involved in tau acetylation have been observed (Min et al., 2015), but these abnormalities are both context and disease specific. For example, p300/CBP HAT activity is increased in frontotemporal lobar degeneration patients (Chen et al., 2020), but lowered in the F2 area of the frontal cortex and hippocampus of AD patients compared to aged controls (Schueller et al., 2020). Tau acetylation at K174, K274, and K281 has been shown to promote cognitive and synaptic defects and impaired hippocampal long-term potentiation (Min et al., 2015; Tracy and Gan, 2017). K280 acetylation has distinct pathological signature markings in different tauopathies and was absent in control brain tissue or cultured wild-type neurons, showcasing the disease-specific nature of K280 acetylation (Irwin et al., 2012). Interestingly, tau isoforms have different propensities to undergo lysine acetylation, with auto-acetylation occurring more prominently in the 2nd and 4th lysine-rich MTBRs, which induced proteolytic tau cleavage and generated distinct N- and C-terminal fragments (Cohen et al., 2016). This group found that auto-acetylation of full-length tau 0N4R leads to production of distinct tau fragments of 43, 38, 17, and 12 kDa tau fragments, a significant reduction in full-length protein, and an approximately 10-fold difference in tau-K18 fragmentation.

Ubiquitination is another covalent modification of proteins on lysine residues that targets proteins for degradation by the 26S proteasome (Stringer and Piper, 2011) and the autophagy-lysosome pathway (Mukhopadhyay and Riezman, 2007; Nathan et al., 2013). It was found that 17 out of a total of 44 lysine residues of tau were ubiquitinated, with the majority found in the MTBR (Munari et al., 2020). Ubiquitin has been found in aggregated tau extracted from tauopathy human brain tissues (Bancher et al., 1991; García-Sierra et al., 2012). Strong association has been found between ubiquitin and an early truncation event at D421 (García-Sierra et al., 2012). These findings suggest interrelationships between phosphorylation and ubiquitination of tau in its aggregated pathology.

SUMOylation is the transfer of a ubiquitin-like protein SUMO to the terminal amino group of lysine side chains and plays roles in a wide range of neurodegenerative diseases (Henley et al., 2014; Anderson et al., 2017). In particular, monoSUMOylation *in vitro* occurs at the K340 in R4 and inhibits tau ubiquitination and subsequent proteasome-dependent degradation, potentially inducing tau accumulation and aggregation (Luo et al., 2014). It was found that 70–75% of lysosomes in inclusion body-positive oligodendrocytes of patients with multiple system atrophy and PSP were SUMO-1 positive with tau, suggesting a role in autophagy-lysosome pathways (Wong et al., 2013).

Glycation (or non-enzymatic glycosylation) is a PTM in which sugars or sugar-derived metabolites are covalently attached to side chains of lysine residues (Li et al., 2012; Rungratanawanich et al., 2021). Glycation processes also involve a set of heterogenous modifications that lead to advanced glycation end-products, which are irreversible cross-links between glycated and non-glycated proteins with relevance to age-related diseases (Sasaki et al., 1998; Simm et al., 2015). Thirty-two lysine residues of tau have been identified to be glycated *in vitro* (Nacharaju et al., 1997; Liu et al., 2016). Many sites are detected in both 3R and 4R, but the presence of K280 and K281 in only 4R isoforms indicate that 4R isoforms (and specifically 2N4R which has the highest number of K residues) with glycation may be related to the higher tendency of 4R isoforms to aggregate.

O-glycosylation is an example of a PTM that has been hypothesized to protect tau from phosphorylation (Liu et al., 2004). O-GlyNAcylation modification of tau inhibits its aggregation (Yuzwa et al., 2012, 2014) and modulates the formation of tau paired helical filaments (Arnold et al., 1996).

While there is evidence for extensive tau PTMs of differing types, the specific roles of these modifications on proteolytic processing of tau is not well known. It will be important in future studies to assess how PTMs may influence tau proteolysis in generating neurotoxic tau fragments.

## Tau Mutations Affect Isoform Ratios

**FIGURE 3:**
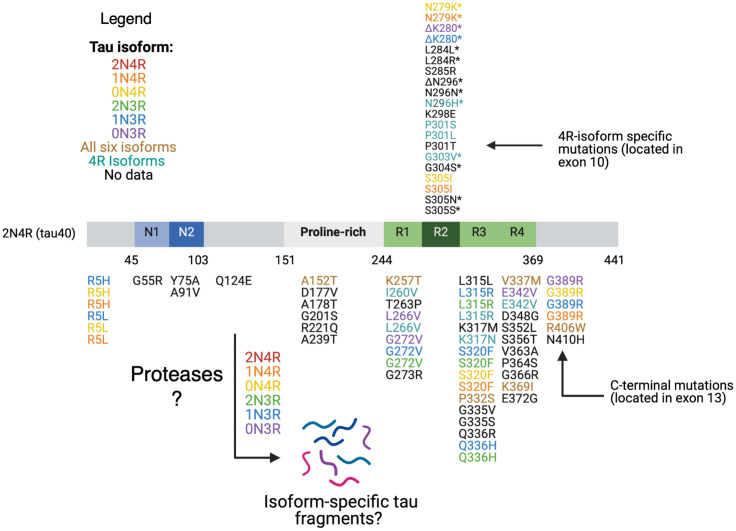
Tau mutations. Tau mutations among parent tau isoforms are illustrated. MTBR tau missense and deletion mutations located in exon 10 and in exons 9,10, 11, and 12 are found in all six isoforms (R1, R2, and R3) and in only 4R isoforms (R2). The mutations are mapped against 441 aa 2N4R isoform. Reported mutations are can be found associated with familial FTDP-tau and other related tauopathy disorders based on evidence from the literature as follows: mutations are colored coded based on their presence in each of the six tau isoforms, in all six isoforms, in 4R isoforms, or no data on isoform(s) containing the mutation (Wolfe, 2009; Rossi and Tagliavini, 2015; Strang et al., 2019). Mutations with a * in exon 10 are associated with alterations in exon 10 splicing and relative isoform expression levels (Wolfe, 2009; Rossi and Tagliavini, 2015). Presently, the details of what tau fragments are generated from each wild-type or mutant tau isoform has yet to elucidated. It will be essential to assess whether 4R-isoform specific mutations generate different and/or similar fragments compared to normal 4R-isoforms, mutant 3R-isoforms, and normal 3R-isoforms.

Over 50 different pathogenic MAPT missense, silent, and intronic mutations have been reported to be present in different sporadic tauopathies such as PSP, CBD, PiD, and FTLD-tau (frontotemporal dementia with parkinsonism caused by MAPT mutations; D’Souza et al., 1999; D’Souza and Schellenberg, 2005; Goedert et al., 2017; Strang et al., 2019). The majority of tau mutations are clustered in the microtubule binding repeat domains of tau (Figure 3). The mutations are composed of two main classes.

The first class includes missense and deletion changes in the coding region and induces the ability of tau to aggregate and form filaments (Arrasate et al., 1999; Goedert et al., 1999; D’Souza and Schellenberg, 2005). This class of mutations includes G272V, Δ280K, P301L, V337M, R406W which have all been found in FTLD-tau patients and caused moderate decreases in microtubule interaction and stabilization (Barghorn et al., 2000; D’Souza and Schellenberg, 2005). P301L particularly increases aggregation potential and largely consists of mutant 4R-tau, with only small amounts of normal 4R- and 3R-tau (Izzu et al., 2000; D’Souza and Schellenberg, 2005).

The second class of mutations affect alternate slicing of MAPT transcripts. These mutations mostly affect exon 10 splicing, and include N279K, L284L, S305N, S305S, Δ296N, and N296H (Goedert et al., 2000; Miyamoto et al., 2001). These specific mutations alter exon 10 splicing and result in an increase in the ratio of 4R- to 3R-tau, indicating that 4R-tau is more toxic (Hutton et al., 1998). In an examination of autopsy material available from mutation carriers, there was an increase in the 4R/3R ratio (Spillantini et al., 1998; Goedert et al., 1999). While it remains unclear fully as to how this ratio leads to aberrant microtubule function, several hypotheses are being examined. One proposed mechanism is that 4R- tau binds to MTs with higher affinity due to its extra microtubule binding domain and displaces 3R-tau, resulting in excess free 3R-tau (Hong et al., 1998; D’Souza and Schellenberg, 2005). Alternative proposals suggest there are discrete MT binding sites for 3R and 4R tau (Goode et al., 2000; Makrides et al., 2004), and the synthesis of excess 4R tau results in saturation of the 4R sites, causing an excess of free 4R tau, which can aggregate (D’Souza and Schellenberg, 2005). This has been shown to occur in cell culture experiments overexpressing 4R tau (Lu and Kosik, 2001). One mutation at position 280, Δ280K, is unique in that it involves deletion of a lysine (mutation at exon 10) and reduces the 4R/3R ratio via ablation of exon 10 splicing (Van Swieten et al., 2007; Momeni et al., 2009).

## Tau Fragments Drive Memory Deficits and Toxicity in Transgenic Animal Models

Tau proteolytic fragments have been shown to drive memory deficits and toxicity in tau animal models of neurodegenerative tauopathies. Numerous tau fragments induce tau pathology and behavioral deficits in *in vivo* animal models of tauopathies (Table 1), and induce *in vitro* cellular toxicities (Table 2).

**TABLE 1:** Tau fragments in *in vivo* models displaying memory deficits and/or cellular toxicity.

Tau fragment	Description of transgenic (Tg) mouse, and transgenes	Memory and behavioral deficits: observed, none, or unknown	Toxicity mechanisms: observed, none, or unknown	Associated proteases	References
1–314 (Δtau314, 35 kDa)	rTg4510 mice expressing tau^*P301L*^, TCP35 tau fragment (35 kDa), also known as **Δ**tau314, compared to wild-type (wt) tau	Memory deficit observed and correlated with levels of TCP35 tau fragment (**Δ**tau314)	Impaired synaptic transmission. glutamate receptor dislocation, hippocampal neuronal loss	Caspase-2 cleaves at D314-L315 (Zhao et al., 2016)	Zhao et al., 2016
187–441 (C-terminal 35 kDa fragment)	Tau35 mice model expressing a 35 kDa tau fragment without mutations and under control of the human tau promotor	Memory deficit and motor dysfunction observed	Increased tau phosphorylation, activation of GSK3b kinase, compromised synaptic function, and impairment of autophagy and lysosome- mediated degradation	Unknown at G186-E187	Bondulich et al., 2016
1–255 and 1–368	Tg mice expressing P301S display tau fragments N255 and N368, generated by AEP.	Memory deficit observed	AEP is up-regulated, and responsible for synapse loss and cognitive deficits shown by AEP knockout.	AEP cleaves at N255-V256 and N368-K369	Zhang et al., 2014
151–421 (Δtau)	TAU62 Tg mice co-expressing 3R tau 151–421 (**Δ**tau) crossed with a 383 aa 4R tau with a P301S mutation (P301SxTAU62), Also studied TAU62 mice crossed with ALZ17 Tg mice expressing wild-type 4R tau AIZ717xTAU62	Memory deficit and motor abnormalities observed in all crossed mice. These included gait ataxia, tremor, and hindlimb reflex deficits	Disruption of axonal transport, mitochondria, Golgi apparatus, and synaptic proteins; co- expression led to severe paralysis within 3 weeks and was rescued by cessation of tau_151–421_ expression	ADAM-10 cleaves nearby at 1151-A152 (Henriksen et al., 2013), Caspase-2 cleaves at 0421-S422 (Zhao et al., 2016)	Ozcelik et al., 2016
45–230 (17 kDa)	Tg mice expressing 17 kDa tau 45–230, compared to wt mice	Memory and learning abnormalities observed, enhanced fear response (fear conditioning test) and longer time in the morris water maze test.	Tau 45–230 expression resulted in synaptic loss and alterations in NMDA receptor subunits	Calpain-1 cleaves at K44-E45 (Yang and Ksiezak-Reding, 1995), Calpain 1- and 2- cleave at R230-T231 (Garg et al., 2010)	Lang et al., 2014
243–441 (Tau-CTF24, 24 kDa)	Tg601 mouse overexpressing wild-type human tau (2N4R) that developed hyperphosphorylated- tau	Memory deficit observed	Accelerated intracellular propagation of tau, reduced capacity for MT assembly compared to tau441	Calpain 1 cleaves at R242-L243 (Matsumoto et al., 2015)	Park and Ferreira, 2005; Garg et al., 2010; Matsumoto et al., 2015

*This table displays tau fragments studied in transgenic mice models that display memory deficits and/or cellular toxicities, with the indicated reference citations. These studies demonstrate that transgenic mice models are useful to the field to study behavioral and memorial abnormalities involving tau fragments.*

**TABLE 2:** Tau fragments in *in vitro* models displaying cellular toxicity.

Tau fragment	Description of *in vitro* model	Toxicity mechanisms of tau	Associated protease	References
1–44	Cerebellar granule cells from 8-day rats or from 6-day-old ERK1^–/–^ mice	Cell death involving extrasynaptic NMDAR excitotoxicity + ERK1/2 pathway	Calpain-1 cleaves at K44-E45	Amadoro et al., 2006; Yang and Ksiezak-Reding, 1995
26–44	Cerebellar granule cells from 8-day rats or from 6-day-old ERK1^–/–^ mice	Cell death involving extrasynaptic NMDAR excitotoxicity + ERK1/2 pathway	Caspase-3 cleaves at K25-Q26, Calpain-1 cleaves at K44-E45	Amadoro et al., 2006; Yang and Ksiezak-Reding, 1995
26–230	Adenoviral expression of tau 26–230 in primary neuronal cells, and differentiated neuroblastoma cells undergoing apoptosis by BDNF withdrawal or treatment with staurosporine	NMDA-mediated neurotoxicity	Caspase-3 cleaves at 025–026, Calpain-1 and -2 cleave at R230-T231	Corsetti et al., 2008
1–156	Cerebellar granule cells obtained from 8-day rats or from 6-day-old ERK1/mice	Unknown	Thrombin cleaves nearby site at R155-G156	Amadoro et al., 2006; Arai et al., 2005
1–255	Primary neurons transfected with adeno- associated viruses encoding tau 1–255	Tau aggregation into PHF with a mixture of tau fragments	AEP cleaves at N255-V256	Zhang et al., 2014
1–368	Primary neurons transfected with adeno- associated viruses encoding tau 1–368	Tau assembly into filamentous structures and PHF, triggered apoptosis	AEP cleaves at N368-K369	Zhang et al., 2014
256–368	Primary neurons transfected with adeno- associated viruses encoding tau 256–268	Tau assembly into filamentous structures and PHF, triggered apoptosis	AEP cleaves at N255-V256 and N368-K369	Zhang et al., 2014
256–441	Primary neurons transfected with adeno- associated viruses encoding tau 256–441	Tau PHFs	AEP cleaves at N255-V256	Zhang et al., 2014
369–441	Primary neurons transfected with adeno- associated viruses encoding tau 369–441	Tau aggregation into PHFs with a mixture of fragments	AEP cleaves at N368-K369	Zhang et al., 2014
243–441 (Tau-CTF24, 24 kDa)	N2a cells expressing Tau-CTF24 and Tau- FL and treated with Thioflavin S to measure aggregation SH-SY5Y cells expressing Tau-CTF24 and exposed to various seeds including heparin-induced assembled Tau, Tau- CTF24, or sarkosyl-insoluble pellets from human tauopathies	Higher aggregation, reduced MT stabilization and MT bundling function in N2a cells In SH-SY5Y cells, Tau-CTF24 showed lower seeding than Tau-FL, but Tau-CTF24 showed greater aggregation to various seeds in the ppt fraction compared to Tau- FL	Calpain 1 cleaves at R242-L243	Matsumoto et al., 2015
1–391	HEK-293FT cell expression of tau fragment	Significant insoluble tau in cells that expressed 1–391, enhanced aggregation due to deletion of last 50 aa	Unknown at E391-I392	Gu et al., 20
1–421	HEK-293FT cell expression of tau fragment	Tau 1–421 induced mitochondria fragmentation and elevated oxidative stress levels (Quintanilla et al., 2009)	Caspase-1, -3, -6, - 7-, and -8 cleave at D421-S422	Gamblin et al., 2003; Quintanilla et al., 2009; Gu et al., 2020
51–391	HEK-293FT cell expression of tau fragment	Enhanced aggregation due to deletion of last 50 aa	N/A at T50-P51,unknown at E391-I392	Gu et al., 2020
51–421	HEK-293FT cell expression of tau fragment	No significant increase in ratio of RIPA insoluble/soluble tau in cells that expressed tau 51–421	N/A at T50-P51, Caspase -1, -3, -6, - 7-, and -8 cleave at D421-S422	Gamblin et al., 2003; Gu et al., 2020
51–441	HEK-293FT cell expression of tau fragment	No significant increase in ratio of RIPA insoluble/soluble tau in cells that expressed tau 51–441	N/A at T50-P51	Gu et al., 2020
151–391	HEK-293FT cell expression of tau fragment	High self-aggregation capacity	ADAM-1 O cleaves nearby at I151-A152, unknown at E391-I392	Gu et al., 2020
151–421	HEK-293FT cell expression of tau fragment	Increases ratio of RIPA-buffer insoluble/soluble tau in cells that expressed tau 151–421, deletion of last 20 aa decreased ratio slightly	ADAM-10 cleaves at 1151-A152, Caspase -1, -3, -6, - 7-, and -8 cleave at D421-S422	Gamblin et al., 2003; Gu et al., 2020
151–441	HEK-293FT cell expression of tau fragment	Increase in ratio of RIPA-buffer insoluble/soluble tau in cells that expressed tau 151–421	ADAM-10 cleaves nearby at I151-A152	Henriksen et al., 2013; Gu et al., 2020
153–441 (Tau-A)	Serum based assay of tissue from confirmed AD post mortem brains, also Tg4510 mice expressing a P301S mutation	Inverse correlation with MDRS, indicating it is related to loss of cognitive ability, elevated 1Ox in Tg4510 mice compared to control mice	ADAM-10 cleaves at A152-T153	Henriksen et al., 2013
231–391	HEK-293FT cell expression of tau fragment	Low expression of tau 231–391, which may lead to undetectable aggregation of tau 231–391, deletion of last 50 aa did not enhance ratio	Calpain 1–2 cleaves at R230-T231, unknown at E391-I392	Garg et al., 2010; Gu et al., 2020
231–421	HEK-293FT cell expression of tau fragment	Increase in ratio of RIPA-buffer insoluble/soluble tau in cells that expressed tau 231–421	Calpain 1–2 cleaves at R230-T231, Caspase-1, -3, -6, - 7-, and -8 cleave at D421-S422	Gamblin et al., 2003; Garg et al., 2010; Gu et al., 2020
231–441	HEK-293FT cell expression of tau fragment	Increase in ratio of RIPA-buffer insoluble/soluble tau in cells that expressed tau 231–441	Calpain 1–2 cleaves at R230-T231 (Garg et al., 2010)	Garg et al., 2010; Gu et al., 2020
1–402 (Tau ΔCasp6)	Post morterm brain samples from affected AD temporal and frontal cortex	Neotope antibody to caspase-6 cleaved tau detected intracellular tangles, extracellular tangles, neuropil threads, and neurotic plaques found in temporal and frontal cortex	Caspase 6 at D402-T403	Guo et al., 2004

*This table includes tau fragments studied *in vitro* neuronal models that displayed neurotoxic molecular mechanisms. Some overlap among fragments studied *in vivo* transgenic mice models and *in vitro* neuronal models exist, such as tau 1–255 and tau 1–368 studied in Zhang et al. (2014), Tau 151–421 studied in Ozcelik et al. (2016), Gu et al. (2020), Tau 243–441 (Tau-CTF24) studied in Matsumoto et al. (2015), and Tau 26–230 studied in Corsetti et al. (2008).*

In rTg4510 mice expressing human mutant tauP301L, levels of the 35 kDa tau fragment, also known as Δtau314, correlates with memory deficits (Zhao et al., 2016). The rTg4510 mice expressed transgenic tauP301L driven by a Ca^2+^/calmodulin kinase II promoter system in the forebrain (Ramsden et al., 2005). Memory deficits are observed between 2 and 3 months of age, several months before the loss of synapses or neurons occurring at 4 to 9 months (Ramsden et al., 2005; Santacruz et al., 2005). Blockade of the formation of Δtau314 by mutagenesis of the caspase-2 cleavage site at D314E resulted in improved memory deficits, which supports involvement of Δtau314 in the behavioral deficit (Zhao et al., 2016). Fluorescence and sedimentation assays compared fibrillation of Δtau314, full-length, and tauP301; Δtau314 showed the lowest propensity to form fibrils, suggesting that there is a different mechanism by which it impairs brain function.

A study of another 35 kDa C-terminal tau fragment was conducted without mutations by expression in Tau35 transgenic mice, and results showed that this 35 kDa tau fragment is sufficient to result in cognitive deficits (Bondulich et al., 2016). Tau35 mice express the C-terminal half of wild-type human tau (aa 187–441) fused at the C-terminus of the haemagglutinin tag and under control of a human tau promoter and targeted to a *Hprt* locus. Transgene expression in Tau35 mice is only approximately 7% of the total amount of tau and thus non-physiological functions of over-expressed tau were avoided. Importantly, the 35 kDa tau fragment is present in human tauopathies and provides a model for disorders such as AD, PSP, and CBD. In the study, Tau35 mice exhibited age-related limb clasping with an incidence rate of 5% by 4 months, an increase to 25% by 5–6 months, and all mice showing paresis by 18 months. At 4 months, Tau35 mice exhibited a kyphosis index (KI) of 4.5, which decreased to 3.4 at 8 months and 2.9 by 14 months, indicating a reduction in KI demonstrates progressive age-related kyphosis. These mice also exhibited molecular deficits such as increased tau phosphorylation, activation of GSKβ, and impairment in autophagy and lysosomal-mediated degradation.

Another transgenic mice model looked at the role of arginine endopeptidase AEP (*Lmnn* gene) in synaptic function and behavior by crossing Lgmn^–/–^ mice with P301S-transgenic mice to knock out AEP (Zhang et al., 2014). At 6 months of age, tau P301S-transgenic mice displayed overt reductions in synapses compared to wild-type and Lgmn^–/–^ mice. P301S/Lgmn^–/–^ mice showed ameliorated synapse lose compared to tau P301S-transgenic mice, indicating that *Lgmn* knockout prevents impairment in synaptic function caused by the tau mutation. Tau is cleaved by AEP in an age-dependent manner. At the age of 8 months, tau is cleaved by AEP when AEP expression is high. Memory function of P301S and P301S/Lgmn^–/–^ mice was also tested using the Morris water maze. Tau P301S/Lgmn^–/–^ had greatly reduced learning deficits and improved memory retention compared to tau P301S-transgenic mice. AEP mediates neurotoxicity by generating at N255 and N368, cleaving two toxic fragments tau 1–255 and tau 1–368, confirmed by injection with AAVs encoding tau P301S and uncleavable AAV-tauP301S/N255A/N368A. TauP301S/N255A/N368-expressing mice showed improved memory indicated by the decreased latency to find the platform in the water maze test and increased percentage of time in the target quadrant. Thus, synaptic function is preserved in mice expressing non-cleavable tau P301S and cleavage of tau is required for the effects of AEP (Zhang et al., 2014).

Another group looked at the interaction between truncation and full-length human tau by generating an inducible mouse line (TAU62) expressing tau 151–421 (239 aa, human 3R) with a P301S tau mice (383 aa, human 4R) to produce the P301SxTau62 transgene mouse model (Ozcelik et al., 2016). Tau62 transgenic mice are generated by co-injection of two Thy 1.2 minigene-based constructs, which are obtained by inserting a tetracycline controlled transcriptional silencer (tTS) element complementary DNA. In the presence of doxycline tau151–421 (Δtau) is expressed. In the absence of doxycycline, tTS binds to tetracycline-responsive element on the Thy1.2 minigene, preventing expression of tau151–421 (Δtau). Interestingly, P301SxTAU62^*on*^ mice showed a drastic motor phenotype starting with gait ataxia at 9 days all the way to severe palsy by 3 weeks of age, which was recovered after 151–421 expression was halted at 3 weeks of age. Homozygous P301S tau mice developed immobilizing limb paralysis at approximately 5–7 months of age. P301SxTAU62 mice also displayed pretangle pathology and axonal transport deficiency, but many of these were also reversible with the cessation of tau 151–421 expression. Another transgenic mice that co-expressed Δtau and full-length 4R human wt tau (ALZ17xTAU62 mice; Ozcelik et al., 2016) displayed nerve cell dysfunction, as well as motor palsy similar to the P301SxTAU62 mice. These findings demonstrate modified behavioral and cellular outcomes of Δtau with and without other tau forms. Heterogeneity in tau fragment forms influences resultant deficits.

Tau 45–230 has also been studied in transgenic mice models (Lang et al., 2014). Generation of Tau 45–230-GTP transgenic mice was conducted by injecting the pronucleus of a single-cell fertilized C57BL/6J mouse embryo with the tau 45–230 transgene under control of the Thy 1.2 promoter. Similar to other transgenic studies, genotyping of tail biopsies by PCR and southern blot were done in order to identify the transgene positive founder mice. Neuronal loss was significant higher in 9- and 12-month old Tg tau 45–230 mice compared to wild type controls. Levels of synaptophysin, a synaptic vesicle membrane protein, were significantly decreased as early as 6 months after birth in the transgenic mice compared to the wild type. Similar significant decreases were found in the NR2A and NR2B subunits of the NMDA receptors in the Tg tau 45–230 mice at the ages of 6 and 9 months. Tg tau 45–230 mice showed enhanced freezing behavior in fear tests. In the Morris water maze test, the tau_45–230_ mice displayed mild memory defects compared to their age-matched wild type controls.

Another fragment called tau-CTF24 (243–441, 24 kDa) was increased with aging in a tauopathy model mice (Tg601) and was found to be produced by increased calpain activity in old Tg601 animals (Matsumoto et al., 2015). Tg601 mice express high levels of 2N4R under control of calcium/calmodulin-dependent protein kinase II promoter. Tau-CTF24 was found more abundantly in the brains of old Tg601 mice than in young Tg601 mice. When comparing non-tg mice and Tg601 mice at the same age, larger amounts of Tau-CTF24 were detected in the Tg601 group than in the non-Tg mice group. Similar results were replicated *in vitro* as well. Recombinant tau-CTF24 accelerated heparin-induced aggregation and lost the ability to promote MT. Insoluble tau from diseased AD brains were introduced as seeds into SH-SY5Y cells and a large amount of insoluble tau was formed in cells overexpressing tau-CTF24 than in those expressing full tau. These results suggest that the absence of the N-terminal domain increases aggregation propensity and lowers MT assembly. Furthermore, lysates containing these tau-CTF24 inclusions propagated to naïve tau-expressing cells more efficiently than those containing full length tau inclusion. Importantly, western blots using the TauN, Tau5, Exon10v2, and TauC antibodies found the tau-CTF24 fragment in brain tissues from AD, familial FTLD with N279K mutation, PSP, and CBD, respectively.

It must be realized that numerous tau fragments identified in animal models have unknown toxicity (examples are shown in Table 3). Among tau fragments found in brains from patients with tauopathy conditions, the contribution of each of the different tau fragments with respect to behavioral dysfunctions and neurotoxicity have not yet been fully defined. It will be important to define the full spectrum of tau fragments in tauopathies and define each of their biological activities.

**TABLE 3:** Tau fragments with unknown pathogenic functions.

Tau fragment	Model description	Toxicity mechanisms	Associated protease	References
1–152	Brain tissue samples from control Sprague Dawley rats and from Tg4510 tau transgenic mice overexpressing the tau mutant P301L and in confirmed AD post mortem brain	Unknown	ADAM-10 cleaves at A 152-T153 (Henriksen et al., 2013)	Henriksen et al., 2013
156–441	Brain tissue from frontal or temporal regions of normal human brains and from perfused Adult Wistar rat brains, incubated with and without protease inhibitors	Thrombin found to cleave tau, however, toxic mechanisms of fragment not studied	Thrombin cleaves at R155-G156 (Arai et al., 2005)	Arai et al., 2005
156–209	Brain tissue from frontal or temporal regions of normal human brains and from perfused Adult Wistar rat brains, incubated with and without protease inhibitors	Thrombin found to cleave tau, however, toxic mechanisms of fragment not studied	Thrombin cleaves at R155-G156 and R209-S210 (Arai et al., 2005)	Arai et al., 2005
210–441	Brain tissue from frontal or temporal regions of normal human brains and from perfused Adult Wistar rat brains, incubated with and without protease inhibitors	Thrombin found to cleave tau, however, toxic mechanisms of fragment not studied	Thrombin cleaves at R209-S210 (Arai et al., 2005)	Arai et al., 2005
210–230	Brain tissue from frontal or temporal regions of normal human brains and from perfused Adult Wistar rat brains, incubated with and without protease inhibitors	Thrombin found to cleave tau, however, toxic mechanisms of fragment not studied	Thrombin cleaves at R209-S210 and R230-T231 (Arai et al., 2005)	Arai et al., 2005
124–441	LC-MS/MS spectrometry of peptides from human brain autopsy samples and N1E-115 mouse neuroblastoma cells	None, cells expressing tau 124–441 had increased α- tubulin acetylation, stronger binding to MTs and less sensitive to depolymerization compared to tau 1–441	Unknown	Derisbourg et al., 2015
127–441	LC-MS/MS spectrometry of peptides from human brain autopsy samples and N1E-115 mouse neuroblastoma cells	Unknown	Unknown	Derisbourg et al., 2015

*This table summarizes identified tau fragments with unknown pathogenic functions, and unknown associated proteases. Tau fragments with unknown toxicity mechanisms and unknown associated proteases were identified in human brain and mouse neuroblastoma cells by Derisbourg et al. (2015), using LC-MS/MS, and include tau fragments 127–441, 172–441, 174–441, 224–441, 238–441, 240–441, 259–441, 261–441, 280–441, 306–41, 309–441, 311–441, and 331–441.*

Significantly, evidence for behavioral deficits and cellular toxicity resulting from expression of tau fragments in transgenic mouse models demonstrate that tau fragments participate in cognitive deficits of tauopathies.

## Tau Truncation: N-Terminal and C-Terminal Cleavages Mediate Aggregation

Tau is abnormally truncated at multiple sites, composing NFT and fibrillar aggregate structures in AD and related tauopathies (Ballatore et al., 2007). A wide spectrum of tau proteolytic fragments has been identified and characterized for neurotoxic and biological functions that may participate in tauopathies (Tables 1, 2; Quinn et al., 2018).

Enzyme-mediated truncation of tau at both the N-terminus (binds neural plasma membrane components) and the C-terminus (binds axonal MT) are facilitators of AD pathology. The deletion of the first 150 or 230 aa enhances tau site-specific phosphorylation, self-aggregation, and seeding, but the deletion of the first 50 aa did not produce significant effects. Similar deletion of the last 50 aa also promote self-aggregation and seeding, but not the deletion of the last 20 aa (Gu et al., 2020).

Several novel fragments consisting of tau 1–391, tau 51–441, tau 51–421, tau 51–391, tau 151–441, tau-151–421, tau 151–391, tau 231–441, tau 231–421, and tau 231–391 were expressed in HEK-293T cells and were analyzed for toxicity by lactate dehydrogenase activity in the cell culture medium leaked from the cells, representing toxicity (Gu et al., 2020). These fragments correspond to well-established truncation sites including K44-E45 by calpain-1 (Yang and Ksiezak-Reding, 1995), A152-T153 by ADAM10 (Henriksen et al., 2013), R230-T231 by calpain 1–2 (Garg et al., 2010), caspase 3/6 by D421-S422 (Zhao et al., 2016), and an unknown protease at E391-I392. Tau 1–421 was also studied, which has been previously shown to aggravate mitochondrial fragmentation and elevated oxidative stress levels (Quintanilla et al., 2009). Tau aggregation was tested by overexpression of aforementioned HA-tagged tau fragments in the HEK-293FT cells which were separated by RIPA buffer-soluble and -insoluble fractions and analyzed the ratios of proteins in RIPA buffer-insoluble/soluble tau using western blot developed with anti HA to quantify concentration. Deletion of the first 50 aa did not affect insoluble/soluble tau ratios, regardless of C-terminal truncations, however, deletion of the first 150 aa significantly increased the ratio in Tau441 and Tau421 forms, but not in Tau391 forms. Deletion of the C-terminal 20 aa did not affect ratios of insoluble/soluble tau, however, deletion of the last 50 aa significant increased aggregation regardless of N-terminal truncations except for Tau231 forms. It was also found that oligomeric tau induces tau aggregation in HEK-293FT cells that were treated and analyzed using the same RIPA buffer-insoluble/soluble ratios of truncated tau. Specifically, Tau 151–391 appeared to have the highest self-aggregation activity and binding to oligomeric tau.

N-terminal tau, which interacts with the plasma membrane (Brandt et al., 1995), is secreted and mediates neurotoxicity at synapses (Sayas et al., 2019; Amadoro et al., 2020; Brunello et al., 2020) demonstrated in tauopathy models. NH_2_-truncated tau fragments of 20–22 kDa have been identified as CSF biomarkers of neurodegenerative diseases and have also been found in mitochondria extracted from cryopreserved synaptosomes (Amadoro et al., 2014;, 2020). Aβ oligomers activated the production of 20–22 kDa NH_2_-terminal tau fragments in human SHSY5Y neuroblastoma and rat hippocampal neurons (Uberti et al., 1997; Amadoro et al., 2010). In a similar experiment, sub-toxic doses of tau NH_2_-terminal peptide (26–44 AA long), which is the minimal active moiety of the longer 20–22 kDa tau peptides, was secreted into the parenchyma and provoked presynaptic deficits of glutamate release on hippocampal synaptosomes and altered Ca^2+^ dynamics (Florenzano et al., 2017). This tau 26–44 fragment is derived from a 17 kDa tau 26–230 fragment via cleavage at D25-Q26 by caspase-3 (Corsetti et al., 2008). Tau 26–44 is seen at the lower end of detection in CSF samples containing both normal and elevated total tau levels, however, the most quantifiable tau peptides in CSF contain the mid-region (aa 156–224), likely due to resistance of degradation by proteases (Barthélemy et al., 2016).

Other studies describe C-terminal truncated tau fragments as another mediator of AD pathology. Flow cytometry data showing synaptic terminals immunolabeled with the tau antibody HT7 highlight an increase in 20 kDa tau fragments and tau dimers in AD synapses (Sokolow et al., 2015). Furthermore, only 15–25% of the synaptosomes were positive for intact C-terminal tau or fully functional MTBR, suggesting that a combination of release of different tau peptides from AD synapses and the phosphorylation of those C-terminal truncated fragments exacerbate tau aggregation and synaptic dysfunction Cleavage and propagation of tau may be due to high tau localization in synaptic terminals in the cortex (Sokolow et al., 2015).

Secretion of full-length and proteolytic tau fragments have been observed in a variety of models. Secretion of tau into the interstitial fluid (Yamada et al., 2011), and eventually into CSF or plasma can be used as a biomarker for AD disease progression (Shoji, 2019). Recently, novel endogenous tau fragments have been identified from CSF samples by immunoprecipitation and mass spectrometry (Cicognola et al., 2019). Several endogenous peptides ending at aa 123 or 224 were identified, which were prominent in the CSF.

## Proteases Known to Generate Toxic Tau Fragments

**FIGURE 4:**
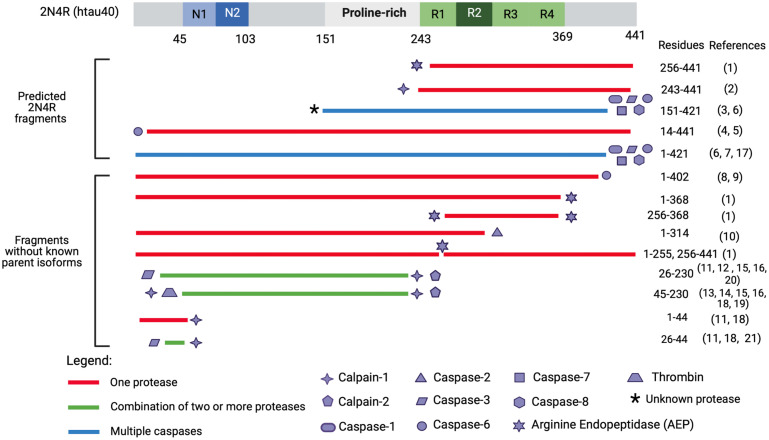
Tau fragments found in *in vitro* and *in vivo* models with pathogenic functions and processing proteases. This figure illustrates a select group of tau proteolytic fragments which have been shown to have to drive neurotoxicity in either *in vitro* or *in vivo* models (correlated with Tables 1, 2) and have a known responsible protease. Precipitating proteases are represented by shape symbols and tau fragments are colored coded based on whether they are cleaved by one or multiple proteases. The top fragments are hypothesized to derive from 2N4R isoform as they extend past aa 410, which is the termination point of the second largest tau isoform, 2N3R. Several fragments from Gu et al. (2020) including tau 51–391, tau 51–421, tau 51–441, tau 151–391, tau 231–391, tau 231–421, and tau 231–441 are not included as they were engineered through recombinant plasmid techniques and have not been identified endogenously. Tau 187–441 and tau 1–391 are excluded as it is unclear which proteases cleaves at G186-E187. Tau 1–156 is excluded as it is unclear if thrombin, which cleaves at R155-G156 (Arai et al., 2005) also cleaves at G156-A157. The * symbol indicates that it is unknown what protease cleaves at the N-terminus of tau 151-421. Tau proteolytic fragments have not been studied to precipitate in up-regulated concentrations from 3R- or 4R-isoforms, and it is unclear whether 3R- or 4R-isoforms more toxic fragments. Reference numbers in parentheses refer to cited articles as follows: (1) Zhang et al. (2014); (2) Matsumoto et al. (2015); (3) Ozcelik et al. (2016); (4) Horowitz et al. (2004); (5) Horowitz et al. (2004); (6) Gamblin et al. (2003); (7) Quintanilla et al. (2009); (8) Foveau et al. (2016); (9) Guo et al. (2004); (10) Zhao et al. (2016); (11) Amadoro et al. (2006); (12) Amadoro et al. (2012); (13) Lang et al. (2014); (14) Afreen et al. (2017); (15) Park and Ferreira (2005); (16) Garg et al. (2010); (17) Matthews-Roberson et al. (2008); (18) Yang and Ksiezak-Reding (1995); (19) Arai et al. (2005); (20) Corsetti et al. (2008); and (21) Florenzano et al. (2017).

An attractive therapeutic approach for attenuating toxicity of tau associated with proteolysis includes inhibiting protease-substrate interactions, reducing protease expression and/or activity, and blocking actions of proteolytic tau fragments. There are a multitude of known tau fragments generated by defined tau cleavage sites, responsible proteases, toxic functions, and are used as biomarkers in disease (reviewed by Quinn et al., 2018). As examples, selected tau proteolytic fragments which have demonstrated roles in neurotoxicity and have at least one known responsible protease for its production are illustrated in Figure 4. Enzymes participating in tau proteolysis consist of caspases, calpains, cathepsins, as well as contributions by the serine proteases A1 (HtrA1), disintegrin, metalloproteinase 10 (ADAM−10), and asparagine endopeptidase (AEP; Quinn et al., 2018).

### Caspase Proteases

Caspase proteases comprise a majority of enzymes involved in proteolysis of tau. Caspases have cysteine in their active site and cleave proteins only after aspartic acid (Asp, D) residues. Caspase members include caspase-2 (Zhao et al., 2016), caspase-3 (Gamblin et al., 2003), and caspase-6 (Horowitz et al., 2004). Over 13 different caspases have been discovered in humans and have been studied extensively (Van Opdenbosch and Lamkanfi, 2019). Many different neurotoxic fragments cleaved by caspases have been detected in mice and cell culture models as well as from patients in serum or CSF. In rTg5410 tau mice, caspase-2 cleaved tau at D134-L315 to result in the production of a N-terminal tau1–314 (Δtau314) fragment. The Δtau314 fragment promotes tau aggregation in dendritic spines (Zhao et al., 2016).

Effector caspase-3, activated by caspase-2, cleaves at D25-Q26 (Corsetti et al., 2008) as well as D421-S422 (Gamblin et al., 2003). Caspase-3 cleavage at D421-S422 results in Tau 1–421 (Tau-C) which is linked to AD and PSP (Gamblin et al., 2003; Zhao et al., 2015). Activation of caspase-3 by a pro-apoptotic activator protein called appoptosin leads to cell death; thus, downregulation of appoptosin can prevent cell death (Zhang et al., 2012).

Caspase-6 cleaves tau at D402-T403 and produces a Tau 1–402 fragment which has been used as biomarker for AD in CSF (Ramcharitar et al., 2013; LeBlanc et al., 2014; Quinn et al., 2018). Caspase-6 activity was found to be elevated in the CA1 of the hippocampus and in the EC-II (LeBlanc et al., 2014) and is extremely abundant in both sporadic (Guo et al., 2004 and familial AD (Albrecht et al., 2009). Notably, increased caspase-6 activity in the anterior olfactory nucleus (which is responsible for mediating signals from the olfactory bulb) is correlated with tau pathology in human AD olfactory bulb brain sections (Foveau et al., 2016). Interestingly, tau pathology is common in the olfactory bulb of AD and Lewis body disease but is minimal or absent in PSP and CBD (Tsuboi et al., 2003).

### Calpain Proteases

Calpains are a second class of calcium-activated cytosolic cysteine proteases that mediate tau proteolysis. These include calpain-1 (Yang and Ksiezak-Reding, 1995; Park and Ferreira, 2005) and calpain-2 (Garg et al., 2010). Dysregulation of calcium homeostasis has been proposed to induce abnormal activation of calpains (Suzuki et al., 2004). Calpain-1 participates in several neurodegenerative diseases including Huntington’s disease and Parkinson’s disease (PD; Siklos et al., 2015) and represents a potential therapeutic target.

Research on calpain has examined the 17 kDA tau fragment spanning residues 45–230 that is produced by calpain-1 via cleavage at K44-E45 (Yang and Ksiezak-Reding, 1995) and calpain-1 and -2 at R230-T231 (Garg et al., 2010). Mouse models overexpressing Tau 45–230 show significantly increased hippocampal death, synaptic loss as early as 6 months, and behavioral abnormalities (Lang et al., 2014). These deficits were also accompanied by alterations in NMDA receptor signaling. More recently, Tau 45–230 mice displayed impaired anterograde and retrograde organelle transport (Afreen et al., 2017). Another cytotoxic fragment, Tau 243–441, was found to be generated by calpain-1; this Tau 243–441 fragment increased in aging mice, propagated to other tau expressing cells (Matsumoto et al., 2015), and initiated degradation of nicotinic acetylcholine receptor subunit α4 (Yin et al., 2016). Calpain-2 has been found to cleave tau at multiple sites that are primarily located in the C-terminal half of the tau protein (Yang and Ksiezak-Reding, 1995).

### Cathepsin Proteases

Cathepsins represent a third class of proteases known to produce neurotoxic tau fragments. Cathepsins are lysosomal proteases utilized for normal protein degradation. However, in many neurodegenerative diseases including tauopathies, abnormal lysosomal leakage results in translocation of cathepsin proteases from the lysosome to the cytosol (Hook et al., 2020). Thus, lysosomal leakage may be a cellular event occurring prior to tau aggregation and NFT or fibrillary aggregate production (Yamashima, 2013; Hook et al., 2020). Several key cathepsins include cathepsins B, D, and L. Cathepsin B is associated with proteolytic tau cleavage and intracellular aggregation; cathepsin B is elevated and accumulates at amyloid plaques of AD brain (Cataldo and Nixon, 1990; Li et al., 1993). Another cathepsin, cathepsin D, participates in tau cleavages occurring at F8-E9, M419-V420, L436-A437, D34-G161, P200-K257, and K267-D358 (Kenessey et al., 1997). Cathepsin L cleaved tau near the C-terminal region of tau, generating two highly amyloidogenic tau fragments. Cathepsin L cleaves at K245-S258, producing tau 258–372 (F1), then subsequently cleaves at V363-P364 to produce tau 258–363 (F2). Cathepsin L then cleaves tau 258–363 at I360-T361 to produce tau 258–360 (F3). F1 did not fully enter the lysosome but rather remained associated with lysosomal membranes (Wang et al., 2009).

### HtrA1, AEP, ADAM-10, and Thrombin

Additional proteases have been found to cleave tau which include human high-temperature requirement serine protease A1 (HtrA1), metalloproteinase 10 (ADAM-10), AEP, and thrombin.

High-temperature requirement serine protease A1 is an ATP-independent serine protease (Quinn et al., 2018) and cleaves between A239 and V399, with preference for leucine, valine, and isoleucine. These cleavages degrade tau and result in 45 different fragments ranging from 1 to 22 aa residues in length. These tau degradative cleavages are associated with low levels of tau and its aggregates in the AD frontal cortex (Tennstaedt et al., 2012). Increased expression of HtRA1 may confer beneficial tau degradation and prevention of NFTs and fibrillar aggregates (Poepsel et al., 2015). HtrA1 is also responsible for cleavage of the lipid transporting apolipoprotein E (ApoE) family, one of the major genetic risk factors for sporadic AD (Chu et al., 2016).

The lysosomal protease AEP, also known as legumain (LGMN), is synthesized as a zymogen (pro-LGMN, 36 kDa) and is auto-catalytically processed by sequential removal of C- and N-terminal pro-peptides to generate a 36 kDa active LGMN enzyme at acidic pH (Dall and Brandstetter, 2016). AEP is active in acidic conditions within lysosomes, but lysosomal leakage results in translocation of AEP into the cytoplasm where it cleaves inhibitor 2 of protein phosphatase 2A (I2PP2A) that leads to increased tau hyperphosphorylation in AD (Basurto-Islas et al., 2018). Cleavage by other proteases is not necessary for AEP processing of tau and AEP cleavage is unaffected by hyperphosphorylation of tau (Zhang et al., 2014). AEP is phosphorylated and activated by serine-arginine protein kinase 2 (Wang et al., 2017) that leads to downstream interaction with tau (Zhang et al., 2014), alpha-synuclein (Zhang et al., 2017), and APP (Zhang et al., 2015). Tau cleavage by AEP occurs at N255-V256 and N368-K369 and results in five prominent tau fragments consisting of tau 1–255, tau 1–368, tau 256–368, tau 256–441, and tau 369–441. Among these fragments, Tau 1–368 and Tau 256–368 were particularly noteworthy for driving apoptosis in primary rat neurons and form PHF (Zhang et al., 2014). AEP is activated during the aging process (Zhang et al., 2014). Aβ oligomers elicited AEP and tau fragmentation in dose-dependent manners in primary rat neuronal cultures, suggesting that Aβ provokes AEP activation (Zhang et al., 2014).

Another protease associated with tau is ADAM-10, a zinc metalloprotease that generates tau 153–441 (Tau A) by cleaving tau at A152-T153 (Henriksen et al., 2013). Upregulation of ADAM-10 cleaves APP into non-amyloidogenic species (reviewed by Andrew et al., 2016) but it is unclear still how membrane-bound ADAM-10 interacts with intracellular tau and whether tau 153–441 has any physiologic relevance to AD (Quinn et al., 2018).

Thrombin is a serine protease that is a component of the coagulation cascade (Chesser et al., 2013) and has been shown to be elevated in microvessels from AD brains compared to microvessels from control brains (Yin et al., 2010). Treatment of HT22 cells with thrombin resulted in formation of thioflavin-S positive tau aggregates within 24 h, followed by increase in cell death at 72 h, but it is unclear how exogenous thrombin alters tau (Suo et al., 2003). However, thrombin has been found to cleave tau (Arai et al., 2005) at multiple arginine and lysine sites including: R155-G156, R209-S210, R230-T231, K257-S258, and K340-S341 and temporally, initial cleavage occurs at R155-G156 (Arai et al., 2005). When tau was phosphorylated by glycogen synthase kinase-3beta, most of the proteolytic processes were inhibited, except for the first cleavage at R155-G156. Additionally, PHFs prepared from AD brains were more resistant to thrombin proteolysis than following dephosphorylation by alkaline phosphatase (Arai et al., 2005). Thrombin-cleaved tau fragments have yet to be found in AD brains and other tauopathies, however.

Alternatively, thrombin may act as a neurotoxin by activating intracellular signaling cascades causing neurite retraction and stimulating apoptosis at high doses (Turgeon et al., 1998; De Luca et al., 2017). Thrombin is also a coagulation factor that plays a role neuroinflammatory diseases such as MS (Göbel et al., 2018). Furthermore, thrombin has been suggested to act as a mediator of vascular dysfunction and inflammation in both the periphery and the central nervous system, contributing to AD by inducing endothelial dysfunction of the blood brain barrier, microglia, astrocytes, and neurons (Iannucci et al., 2020).

### Unknown Proteases for Production of Tau Fragments

It must be realized that while numerous tau fragments have been identified in tauopathy conditions in human brain, animal models, and cellular conditions, the proteases responsible for generating many of these tau fragments are unknown. It will be important to determine the proteases that generate toxic tau fragments, and also compare the relative toxicities of the spectrum of tau fragments.

## The Missing Link: Relationship Among Tau Isoforms, Proteases, and Proteolytic Fragments That Drive Neurotoxicity

Current studies have not provided the full details of which parent tau isoform(s), with or without mutations, serve as the source of specific tau fragments (Figure 3). Rather, identified disease-related tau fragments have been described with respect to the largest 2N4R (hT40) tau isoform (Figure 4). Among identified disease-exacerbating tau fragments, we propose that several fragments belong exclusively to the 2N4R isoform based on the presence of their C-terminus containing residues beyond amino acid 410, which include a C-terminal truncated fragment 1–421, and several N-terminal truncated fragments: 14–441, 51–441, 51–421, 124–441, 151–421, 151–441, 156–441, 231–441, 231–421, 243–441, 369–441, 403–441, and 422–441. Full determination of the mechanisms for proteolytic processing of each tau isoform has been challenging for several reasons consisting of: (1) the 6 tau isoforms share common sequence domains which requires detailed mass spectrometry analyses through multiple strategies to define the isoform origin of tau fragments, (2) the status of phosphorylated or non-phosphorylated tau isoforms and tau-derived fragments as substrates for proteolysis has not been well defined, and (3) the sensitivity of peptide identification methods by immunochemical, mass spectrometry, and others differ greatly. Detailed understanding of the proteases that convert each of the tau isoforms into toxic fragments will be important to develop inhibitors of target proteases that may attenuate production of the most neurotoxic tau fragments that underlie specific tauopathy brain disorders.

## Conclusion

Protease-mediated tau truncation is an important PTM which drives behavioral dysfunction and neurodegeneration in a tau fragment-dependent manner. While numerous tau fragments have been identified, knowledge of the proteolytic steps that convert each parent tau isoform into specific truncated tau fragments has not yet been fully defined. Therefore, the main conclusion of this review is that novel, intensive research is needed to define the details of the proteolytic steps utilized to convert each tau isoform into the most toxic fragments that drive behavioral deficits and tau pathology. Future advancement in understanding of the proteolytic processing mechanisms of tau isoforms can lead to new protease targeted drug strategies to prevent the formation of toxic tau fragments and ameliorate tauopathy disease deficits.

## Author Contributions

BB and VH planned the topics covered and wrote the manuscript. VH guided the overall outline, writing, and editing of the manuscript. Both authors contributed to the article and approved the submitted version.

## Conflict of Interest

The authors declare that the research was conducted in the absence of any commercial or financial relationships that could be construed as a potential conflict of interest.

## Publisher’s Note

All claims expressed in this article are solely those of the authors and do not necessarily represent those of their affiliated organizations, or those of the publisher, the editors and the reviewers. Any product that may be evaluated in this article, or claim that may be made by its manufacturer, is not guaranteed or endorsed by the publisher.
